# Bayesian estimation of *Pseudomonas aeruginosa* viscoelastic properties based on creep responses of wild type, rugose, and mucoid variant biofilms

**DOI:** 10.1016/j.bioflm.2023.100133

**Published:** 2023-06-03

**Authors:** Mohammad Nooranidoost, N.G. Cogan, Paul Stoodley, Erin S. Gloag, M. Yousuff Hussaini

**Affiliations:** a Department of Mathematics, Florida State University, Tallahassee, FL, USA; b Department of Microbial Infection and Immunity, The Ohio State University, Columbus, OH, USA; c Department of Orthopaedics, The Ohio State University, Columbus, OH, USA; d National Centre for Advanced Tribology at Southampton (nCATS), National Biofilm Innovation Centre (NBIC), Department of Mechanical Engineering, University of Southampton, UK; e Department of Biomedical Sciences and Pathobiology, Virginia-Maryland College of Veterinary Medicine, Virginia Tech, Blacksburg, VA, USA

**Keywords:** Biofilm, Viscoelasticity, Biomechanics, Extracellular polymeric slime, Bayesian estimation

## Abstract

*Pseudomonas aeruginosa* biofilms are relevant for a variety of disease settings, including pulmonary infections in people with cystic fibrosis. Biofilms are initiated by individual bacteria that undergo a phenotypic switch and produce an extracellular polymeric slime (EPS). However, the viscoelastic characteristics of biofilms at different stages of formation and the contributions of different EPS constituents have not been fully explored. For this purpose, we develop and parameterize a mathematical model to study the rheological behavior of three biofilms — *P. aeruginosa* wild type PAO1, isogenic rugose small colony variant (RSCV), and mucoid variant biofilms against a range of experimental data. Using Bayesian inference to estimate these viscoelastic properties, we quantify the rheological characteristics of the biofilm EPS. We employ a Monte Carlo Markov Chain algorithm to estimate these properties of *P. aeruginosa* variant biofilms in comparison to those of wild type. This information helps us understand the rheological behavior of biofilms at different stages of their development. The mechanical properties of wild type biofilms change significantly over time and are more sensitive to small changes in their composition than the other two mutants.

## 1 Introduction

Bacterial pathogens and other microorganisms adhere and grow at surface interfaces. This population forms a biofilm, which is a community of adherent microorganisms encased in a self-produced extracellular polymeric slime (EPS). The EPS is complex and composed of extracellular DNA, protein, and multiple species of exopolysaccharides. These EPS components are involved in biofilm development and attachment to a substratum, and they assure structural integrity of biofilm [[1], [2], [3], [4], [5]]. This EPS network is heterogeneous and subject to change over time. Therefore, studying the material properties of biofilm and EPS interactions is fundamental to understanding structural dynamics and developing methods for removing and preventing biofilm-induced infections [[6], [7], [8]].

Mathematical models and numerical schemes have been developed since the 1980s to model the dynamics of biofilm development in order to understand the physics of such bio-organism systems and to predict the growth and development of these communities. These models focus on different spatial and temporal scales, which show the inherent multiscale nature of such biomechanical systems [9]. Wanner and Gujer's 1-D model [10] is amongst one of the first models, which described the dynamics and spatial distribution of bacteria in biofilms. Later, different continuum and individual-based models were developed to deterministically and stochastically investigate biofilm growth and bacteria population within biofilms [11]. Individual-based models were found to be useful tools to simulate mixed-species biofilms [[12], [13], [14]]. Xavier and Foster [15] investigated the competition dynamics between different strains that differ in the level of polymer production and predicted that mixed-strain biofilm tends to have increased polymer production; however, polymer production is not expected to increase indefinitely, and it will stabilize at an intermediate level. Zhang et al. [16]'s multiscale model showed that the biofilm community is a complex system, that its metabolism is coupled to the spatial dependence of external chemical concentrations.

Another important element in biofilm dynamics is the distribution of polymeric components and water content within the biofilm, significantly influencing how biofilm moves under different flow conditions [17]. Computational fluid dynamics can be combined with other mathematical models to investigate biofilm dynamics [18] as well as the effects of flow on biofilm growth on different surfaces and complex geometries [19,20]. Other factors that flow simulations can model are the wettability, elasticity, and antimicrobial properties of surfaces [11]. Cogan and Keener [21] showed that diffusion-driven growth can lead to heterogeneous towers and mushroom-like structures via fluid/structure instabilities modulated by the interaction between differential production and chemical properties of the EPS.

Despite the numerous research studies on biofilm modeling, the quantification and parameterization of biofilm models, how EPS properties affect biofilm structure and maturation, and the interactions between the components of biofilm remain largely unexplored [22]. Typical models of biofilm mechanics fail to quantify the viscoelastic properties of biofilms. Zhang et al. [23] incorporated viscoelastic stresses built in biofilms into their mathematical model and described the interaction between biofilm components and fluid flow. Klapper et al. [6] formulated a mathematical model based on the Jeffrey viscoelastic fluid constitutive law and found that biofilms behave as viscoelastic fluids, demonstrating both the unreversed flow as well as the elastic and viscoelastic recoil. Later, the viscoelastic properties of such biofilms were quantified using creep-recovery and oscillatory frequency sweep tests [24]. Tierra et al. [25] characterized the effects of mechanical parameters of biofilm components in the fluid–biomass interaction and concluded that biofilm's resistance to deformation introduced by flow shear can be largely attributed to its viscosity. It should be noted that all these models assumed simple-constituent EPS.

One very important characteristic of biofilms is their wide variability [26]. This variability is present between samples, where biofilms of the same strain and growth conditions lead to different biofilm structures, due to spatial diversity and heterogeneity [27]. Within a single biofilm, the growth rates, cell densities, and mechanical properties also vary in both time and space, due to gradients established within the biofilm and the diverse EPS production. This variability and the inherent uncertainty of model parameters require a detailed uncertainty analysis. The model parameters can be estimated based on observational data to optimize the mathematical model. Coupling experimental observations with mathematical modeling has been well established in other fields such as meteorology [28], where it is generally referred to as data assimilation (DA). These techniques were later used in other fields, such as ecology [29,30], and engineering applications, for example, dielectric elastomers, solid amorphous polymers, and lithium-ion batteries [[31], [32], [33]]. However, this is much less widespread in biomathematics applications, where challenges in estimating relevant parameters are unique [[34], [35], [36]]. For example, one of the most important outcomes in biological DA is to predict and optimize the relevant factors involved in biofilm development so that efficient optimal control targets can be identified by coupling DA with sensitivity analysis [36].

In this paper, we develop a Bayesian framework that assimilates experimental data with linear viscoelastic models to help us estimate the viscoelastic parameters of different *P. aeruginosa* variants that emerge during chronic infections. First, we model biofilm EPS by a viscoelastic Burger model, that consists of a combination of springs and dashpots, representing the elasticity and viscosity of biofilm, respectively. Then, we utilize a Bayesian estimation platform based on a Markov chain Monte Carlo (MCMC) method to estimate the viscoelastic properties of *P. aeruginosa* biofilm variants using experimental data [24]. MCMC methods comprise a class of stochastic techniques which use a set of discrete samples to approximate model parameters as a posterior distribution [37]. We use a deterministic viscoelastic model, with parameters drawn from a distribution, to explore the stochastic nature of this behavior. This stochasticity is due to the parametric variability of biofilm viscoelastic properties.

Biofilm structure and chemistry change over the course of their development, and there is a high variability in biofilm mechanical properties due to their intrinsically heterogeneous and dynamic behavior. These biofilm characteristics, together with the use of different analysis parameters, including timescales of analysis and magnitude of forces applied, along with the unavoidable errors in measurement techniques add uncertainties to the measurement and quantitative analysis of biofilm mechanical properties [24]. Given the variability in biofilm measurements, incorporating uncertainty is very important for understanding model predictions. Therefore, this line of research will help guide future studies focusing on different biofilm variants at different stages of formation and lead to better predictive modeling.

## 2 Materials and methods

### 2.1 Bayesian data assimilation framework

We designed a Bayesian data assimilation framework to find the rheological estimates of biofilm EPS based on theoretical viscoelastic models and experimental data. We systematically assimilated experimental data to better estimate the relevant parameter values involved in the biofilm rheology and quantify the uncertainty in their measurement and prediction as a probability. Unlike classical statistics and non-Bayesian parameter estimation approach, Bayesian methods provide distributions for estimated parameter sets based on the knowledge we have from experimental data, the prior information we have from parameters of interest, and the mathematical model structure.

**Fig. 1:**
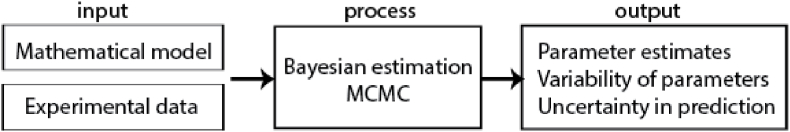
The flowchart of our Bayesian framework.

Our Bayesian-based algorithm is briefly presented in Fig. 1. First, we assembled the input, including the theoretical model and experimental data for our Bayesian estimation toolbox. Our theoretical model was parameterized using a linear viscoelastic model that describes the viscoelastic response of biofilm EPS under constant shear stress during a creep-recovery test. The experimental data were obtained from the experiments on creep-recovery measurements of different biofilm variants at different stages of their formation [24]. Then, using our Bayesian-based parameter estimation technique, which is explained in the next sections and Appendix A, we computed the distributions of estimated values for each biofilm's viscosity and elasticity. These distributions give us insight into the uncertainty and variability of each model parameter, as well as the prediction of error variance for future measurements.

### 2.2 Theoretical viscoelastic model

Viscoelasticity is a mechanical property that characterizes the rheological behavior of a material. It exhibits both the viscous and elastic characteristics of a substance when they undergo mechanical deformation. Linear viscoelastic models have been used to describe polymeric solutions [38] and to quantify viscoelastic properties of polymeric substances such as biofilm matrix [39], where polymeric substances undergo a small strain and where we can assume there is a linear relationship between stress and strain. These models are structured by various combinations of linear spring and dashpot elements, representing elasticity and viscosity, respectively. These elasticity parameters characterize biofilms’ tendency to reform their shape after being stretched under stress, while viscosity characterizes biofilm resistance to deformation.

**Fig. 2:**
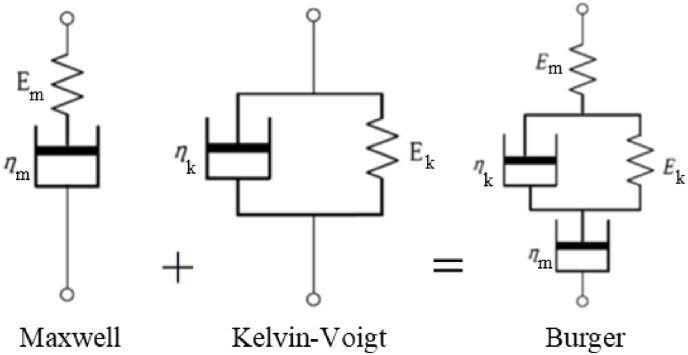
The arrangement of spring and dashpot components in Maxwell, Kelvin-Voigt, and Burger models.

The numbers and arrangements of spring and dashpot elements can be altered to provide different linear viscoelastic models, such as the Maxwell, Kelvin-Voigt, and Burger models. The Maxwell and Kelvin-Voigt models consist of one spring and one dashpot connected in series and in parallel, respectively. The Burger model contains a spring and a dashpot in series (Maxwell compartment) connected to a spring and a dashpot in parallel (Kelvin-Voigt compartment), as shown in Fig. 2. *E* denotes spring coefficient, and *η* denotes dashpot coefficient. The underscripts *m* and *k* represent the Maxwell and Kelvin compartments, respectively.

The Maxwell model is accurate in modeling the instant elastic strain increase during loading and the elastic strain decrease right after unloading stress; however, it captures neither the time-dependent recovery nor the decreasing strain rate of substance under a creep-recovery test. On the other hand, although the Kelvin-Voigt model precisely shows the time-dependent recovery, it does not demonstrate the instant strain during loading and unloading. Thus, it is clear that a mix of both models is needed to properly describe the viscoelasticity of complex rheological substances.

After exploring different linear viscoelastic models, we chose to use the Burger model which is a combination of the Maxwell and Kelvin-Voigt models. There are several advantages to using this model: Firstly, we were able to analytically solve for the strain and therefore, allowing us to run our data assimilation scheme 10, 000, 000 times to estimate our parameter estimates with high precision. Secondly, this relatively simple model helped us avoid over-fitting, which is a common problem in models with many parameters and modest amount of data.

In the Burger model, the instant increase in elastic strain at the beginning of the creep test, which fully recovers after unloading shear stress, is characterized by the Maxwell spring (*E*_*m*_), while the strain rate at the end of the creep test is described by the Maxwell dashpot (*η*_*m*_). The Kelvin-Voigt spring (*E*_*k*_) and Kelvin-Voigt dashpot (*η*_*k*_) are accountable for the gradual increase and decrease in strain during creep and recovery tests. The constitutive equation for a Burger model can be derived based on linear spring and dashpot equations:

(1) $$\left(\frac{\eta_{m}\eta_{k}}{E_{m}E_{k}}\right)\ddot{\sigma }+\left(\frac{\eta_{m}}{E_{k}}+\frac{\eta_{m}}{E_{m}}+\frac{\eta_{k}}{E_{k}}\right)\dot{\sigma }+\sigma =\left(\frac{\eta_{m}\eta_{k}}{E_{k}}\right)\ddot{\epsilon }+\eta_{m}\dot{\epsilon }$$

where *σ*, *ϵ*, *E*, and *η* denote the stress, strain, linear spring constant, and linear dashpot constant, respectively. $\dot{\sigma }$ and $\ddot{\sigma }$ are the first and second time derivatives of the stress; while $\dot{\epsilon }$ and $\ddot{\epsilon }$ are the first and second time derivatives of the strain, respectively. The underscripts *m* and *k* represent the Maxwell and Kelvin compartments in the Burger model. Note that, the Maxwell elements (*E*_*m*_ and *η*_*m*_) are an elastic element and a viscous element in series, respectively, and the values associated with these elements can be isolated and calculated directly through experiments, while the Kelvin elements (*E*_*k*_ and *η*_*k*_) are in parallel and interact and do not have direct, measurable interpretations. They are present in our theoretical model to describe the time-independent creep and recovery response of biofilm EPS.

**Fig. 3:**
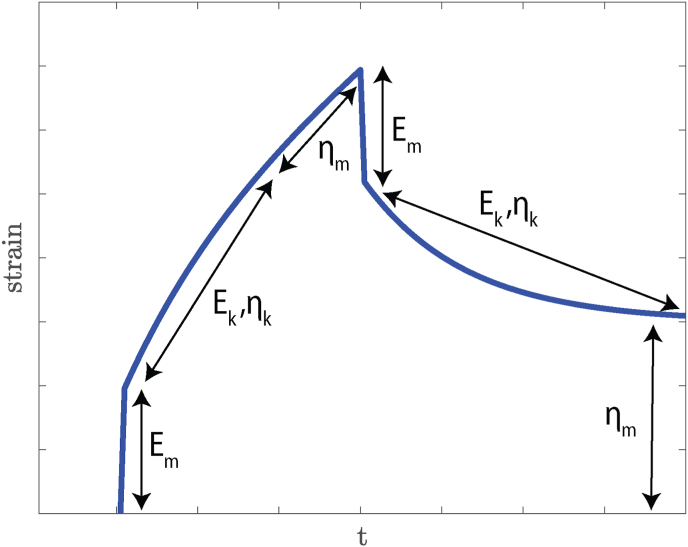
The different stages of a typical biofilm strain response to the creep-recovery test.

We used the aforementioned Burger model to parameterize the rheological response of our biofilms during a creep-recovery test. Creep-recovery response tests are among the standard tests to measure the viscoelastic properties of biofilms and to characterize the time-dependent responses of materials during loading and unloading of constant shear stress [39]. In this mechanical test, a sudden fixed shear stress (*σ*_0_) is applied to biofilm for a specified time period (creep test), and then it is unloaded after a certain time (recovery test). Biofilm responds to this creep-recovery test by deforming in the direction of the applied shear stress. The viscoelastic parameters of biofilm will be extracted based on this deformation and the time-dependent response [40]. The measured local displacement in the direction of the stress is non-dimensionalized by the biofilm thickness and is called strain (*ϵ*). This total strain explained in the context of a spring-dashpot model is essentially the sum of three separate strains: 1) The instant elastic strain which occurs right after loading the constant stress and fully recovers right after unloading. This strain is characterized by the Maxwell spring. 2) The gradual strain response that is due to the Kelvin spring and dashpot. This strain increases gradually under the applied stress during the creep test and will fully recover during the recovery test. 3) The strain due to the Maxwell dashpot. This strain progressively increases during the creep test; however, it will not recover once the stress is unloaded. As a result, at the end of the recovery test, a permanent strain will remain. Our theoretical model has no viscoplastic elements, and the biofilm is loaded under a low constant shear stress (0.5 Pa), which is below the biofilm yield stress. Therefore, it is in the viscoelastic range and will not exhibit any instantaneous viscoplastic strain response related to the viscoplasticity behavior. Fig. 3 shows the different stages of biofilm strain response to a creep-recovery test. The elements that are informed by each stage of the strain response are illustrated in the figure.

In a creep-recovery test, shear stress is constant, and thus stress derivatives are zero. Therefore, equation (1) reduces to:

(2) $$\begin{array}{lll} \left(\frac{\eta_{m}\eta_{k}}{E_{k}}\right)\ddot{\epsilon }+\eta_{m}\dot{\epsilon }=\sigma_{0}, & \text{ if }t<\tau & \end{array}$$

(3) $$\begin{array}{lll} \left(\frac{\eta_{m}\eta_{k}}{E_{k}}\right)\ddot{\epsilon }+\eta_{m}\dot{\epsilon }=0, & \text{ if }t\geq \tau & \end{array}$$

where *σ*_0_ is the constant shear stress during creep test, and *τ* is the time that shear stress is unloaded. At *t* = 0, biofilm experiences an instant elastic strain, therefore, the initial strain can be calculated as $\epsilon \left(0\right)=\frac{\sigma_{0}}{E_{m}}$. The rate of change in the strain at the initial condition is represented by both the Maxwell and Kelvin-Voigt dashpots $\dot{\epsilon }\left(0\right)=\frac{\sigma_{0}}{\eta_{m}}+\frac{\sigma_{0}}{\eta_{k}}$. Solving equations (2), (3)) with these initial conditions leads to:

(4) $$\begin{array}{lll} \epsilon =\frac{\sigma_{0}}{E_{k}}\left(1-e^{-\left(E_{k}/\eta_{k}\right)t}\right)+\frac{\sigma_{0}}{\eta_{m}}t+\frac{\sigma_{0}}{E_{m}}, & \text{ if }t<\tau & \end{array}$$

(5) $$\begin{array}{lll} \epsilon =\frac{\sigma_{0}}{E_{k}}\left(e^{\left(E_{k}/\eta_{k}\right)\tau }-1\right)e^{-\left(E_{k}/\eta_{k}\right)t}+\frac{\sigma_{0}}{\eta_{m}}\tau , & \text{ if }t\geq \tau & \end{array}$$

### 2.3 Experimental data

*P. aeruginosa* is an opportunistic pathogen associated with biofilm-associated chronic infections, specifically in immunocompromised people. *P. aeruginosa* is also considered a model organism for studying biofilms. The EPS of *P. aeruginosa* biofilms is complex, and consists of three different exopolysaccharides: alginate, Pel and Psl, and extracellular DNA and proteins, including CdrA [41].

During chronic infections, *P. aeruginosa* adaptively evolves to form variants that have increased fitness and survival. Of particular interest are the mucoid variants and rugose small colony variants (RSCVs). These colony variants acquire mutations that lead to the overproduction of extracellular matrix components. Genetic mutations lead to the overproduction of alginate in mucoid, and overproduction of Psl, Pel polysaccharides, and the biofilm matrix protein CdrA in RSCVs [42]. Due to the overproduction of these EPS components, both variants have increased biofilm phenotypes compared to the ancestor. We were therefore interested in determining if the overproduction of EPS by mucoid and RSCVs was also associated with changes in biofilm mechanics, relative to the parental wild type strain [24].

To assess this colony-biofilms of *P. aeruginosa* wild type PAO1 and isogenic mucoid variant (PAO1 *mucA22*) and RSCV (PAO1 Δ*wspF*) were analyzed. Sterile nitrocellulose filter membranes (25 mm, 0.45 μm pore size; Milliopore) were inoculated with overnight cultures normalized to OD_600*nm*_ 0.1. Filters were transferred to Pseudomonas isolation agar, and incubated at 37°*C*. Colony-biofilms were transferred to a new plate every 24 h. Colony-biofilms were analyzed days 2, 4 and 6 [24].

At each time point, biofilms were analyzed by uniaxial mechanical indentation and shear rheology [24]. Of relevance to this study, biofilms were analyzed by creep-recovery, using a Discovery Hybrid-2 rheometer (TA instruments) fitted with a 25 mm Smart-Swap sand blasted geometry. Creep-recovery measurements were performed by applying a shear stress of 0.5Pa for 60*s*, followed by a 120*s* recovery [24]. 4 colony-biofilms were analyzed at each timepoint. The experimental conditions of these replicates were consistent, and any variability was intrinsic to biofilm growth and development rather than the experimental design and analysis.

### 2.4 Parameter estimation process

We used the strains calculated by the Burger model (equations (4), (5))) as estimates for our biofilm deformation under shear stress during a creep-recovery test (*σ*_0_ = 0.5 Pa). However, we know that this model, like any other mathematical model, can not predict real experiments perfectly, as there are always errors involved in predicting real-world events. Let's assume our observations are independent and identically distributed (i.i.d), meaning that each observation has the same probability distribution as others and the observations are mutually independent [43]. Note that this assumption is across different observations, not along the creep-recovery test time-series data. We used four observations for each dataset, each consisting of a time-series of strain. Thus, we can assume the errors were normally distributed with standard deviation *γ*, which is a common practice in many engineering and real-world applications [44]. Therefore, the likelihood, the probability of the observed data *y* (measurements of the creep-recovery test), given the model parameters *θ* (elasticities and viscosities) and the error variance *γ*^2^, can be written as:

(6) $$p\left(y|\theta ,\gamma^{2}\right)=\prod_{i=1}^{N}\frac{1}{\sqrt{\left(2\pi \right)}\gamma }e^{-\frac{{\left(x_{i}-y_{i}\right)}^{2}}{2\gamma^{2}}}$$

where *x*_*i*_, and *y*_*i*_ are the *i* th of the *N* model-derived estimate and observed data points, respectively. In this work, *x*_*i*_ are the time series of calculated strain *ϵ* from equations (4), (5)) that is a function of model parameters (*E*_*k*_, *η*_*k*_, *E*_*m*_, and *η*_*m*_). *y*_*i*_ are the measured values of strain over time from the creep-recovery experiments that were explained in the previous section. This likelihood is then incorporated with Bayes’ theorem to calculate the probability of viscoelastic model parameters given the experimental data [45].

A Markov Chain Monte Carlo (MCMC) method [45] was used to construct the target posterior distributions, which are our desired distributions for the viscoelastic parameters. Various MCMC sampling algorithms have been developed over the past decades. Metropolis-Hastings (MH) is one of the classic sampling methods [46] for MCMC Bayesian estimation, that generates sample candidates from a parameter space. These sample candidates are then either rejected or accepted based on the posterior ratio of the new parameter candidate to the previous parameter. Here, we employed a modified MH algorithm which helped us improve the efficiency and speed of our computations by increasing convergence and acceptance rate [47]. The details of our numerical algorithm can be found in Appendix A.

This Bayesian data assimilation framework provides us with estimates and variability of our model parameters, as well as information about the stochastic structure of our data, and the relationship between model parameters. These parameter estimates are formed as distributions of samples that can be used to construct probability density functions. The shape of these probability density functions represents the variability and uncertainty of the parameters. In the context of biofilm EPS viscoelasticity, Bayesian data assimilation helps us have distributions for the viscosity and elasticity in a probabilistic form. The mean of these distributions represents the deterministic value of viscosity and elasticity, showing how viscous and deformable the biofilm is, whereas the variance and shape of the distribution suggest how certain we are in determining the mechanical properties of biofilm. For example, we know biofilms are very heterogeneous and dynamic substances, and a small change in their composition during their early stages of formation may lead to statistically significant (*P* < 0.05) changes in their mechanical properties. Our results, which are discussed in the next section, show that these changes in the mechanical properties of biofilm can be up to two orders of magnitudes. The quantification of this variability helps us understand the biofilm dynamics and develop robust bounds on the uncertainty of our predictions.

## 3 Results

Here, we characterize the viscoelasticity of *P. aeruginosa* biofilms, including wild type (WT) PAO1 and isogenic RSCV and mucoid variant biofilms grown for 2, 4, and 6 days with four viscoelastic parameters (*E*_*m*_, *η*_*m*_, *E*_*k*_, and *η*_*k*_). These viscoelastic parameters and the uncertainty of these parameters were estimated and the error variance was quantified using the creep-recovery experimental data [24] and the Bayesian mathematical platform.

**Fig. 4:**
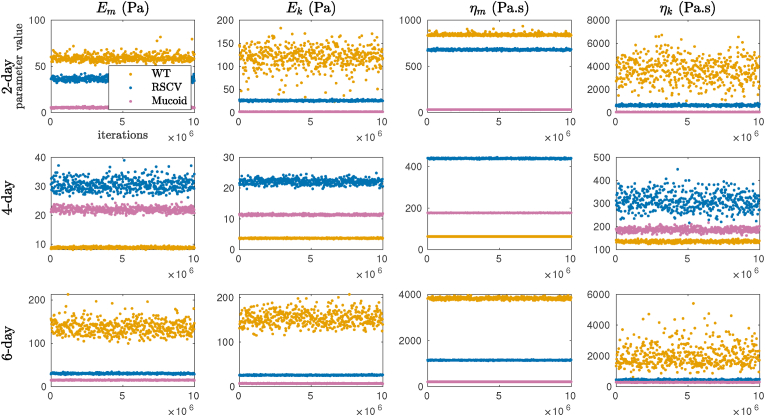
MCMC samples of viscoelastic properties after disregarding the first half of the Markov chains for WT (orange color), RSCV (blue color), and Mucoid (pink color) after 2, 4, and 6 days of formation. (For interpretation of the references to color in this figure legend, the reader is referred to the Web version of this article.)

For this purpose, the four viscoelastic model parameters for each biofilm were sampled from a uniform proposal distribution. First, the MCMC algorithm was run for an initial run with 10, 000, 000 iterations. This is referred to as the “burn-in” time. Then, these results were used for the main run with a second round of 10, 000, 000 iterations. We disregarded the first 10, 000, 000 samples to eliminate the impact of random initial guess on our target proposal distribution and considered the second 10, 000, 000 samples to construct the Markov chains, that equal to the posterior distributions of the parameters (Fig. 4). We observe that the accepted candidates fit in a narrow bound of parameters. However, as shown in the figure, the viscoelastic parameters vary significantly between the three biofilms, and also between different stages of formation (e.g. depending on the age of the biofilm). The high variability of WT viscoelastic parameters is related to the high variability and heterogeneous complexity in the structure of WT biofilms with more uncertainty at the early stages of their formation.

**Fig. 5:**
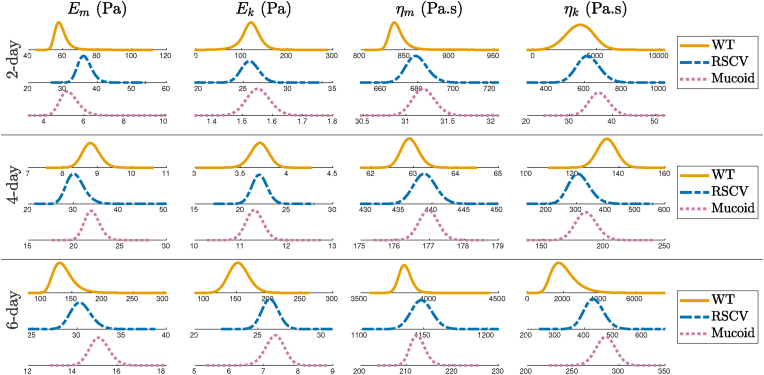
Posterior density distributions of viscoelastic properties for WT, RSCV, and Mucoid after 2, 4, and 6 days of formation. WT 2-day and WT 6-day are highly skewed and have the highest variations in parameters.

Then, we employed a kernel density estimation (KDE) algorithm to calculate the probability densities of posterior distributions. Fig. 5 presents these densities for the three biofilms at different stages of their formation to better visualize how the estimated parameters form a distribution. We observe that these distributions are approximately Gaussian for all parameters, which presents the stochasticity in the physics of biofilm viscoelasticity. Biofilms undergo several chemical and biological processes over the course of their development, and these processes are highly dependent on the state of the system and physical conditions during experiments, which are not fully controllable. Thus, there is an inherent unpredictability in the physical and chemical properties of the biofilm components. The distributions of WT biofilm properties are highly skewed and have the highest relative variations (coefficient of variations) in parameters, especially for Kelvin parameters, which attributes to the heterogeneity and unpredictability of their physics and structure. The WT biofilm is grown from unaltered *P. aeruginosa* and is inherently unpredictable.

**Fig. 6:**
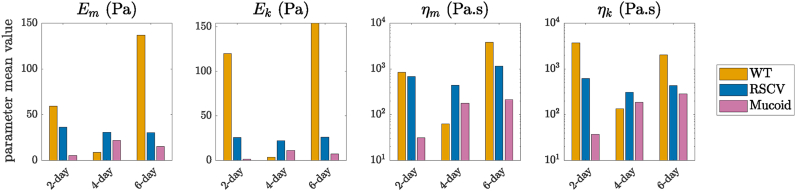
Mean values of estimated viscoelastic properties for WT, RSCV, and Mucoid after 2, 4, and 6 days of formation.

The mean values of the estimated parameters are shown in Fig. 6. The plots for viscosities are on a logarithmic scale as they vary significantly between the three different biofilms and stages of formation. We observe that the Maxwell and Kelvin-Voigt elasticities and viscosities do not follow a similar trend for the three biofilms. Table 1 shows these mean values of the four viscoelastic parameters for each biofilm. The numbers in red are the estimated values of [24] which are presented here for the sake of comparison with our estimations.

**Table 1:**
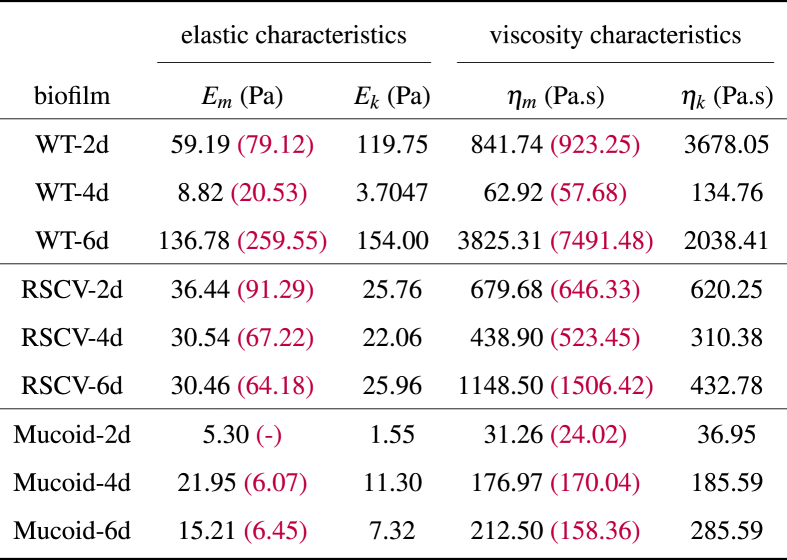
Means of estimated values for viscoelastic parameters of Burger model.

The coefficient of variation (CV) and skewness values for the estimated distributions are listed in Table 2, Table 3, respectively, as measures of variability of the estimated parameters. The coefficient of variance is a statistical measure of the dispersion of data around the mean, whereas skewness is a measure of the asymmetry of posterior distributions about their mean values. The values of the coefficient of variation were calculated by dividing the standard deviations by the mean values, then multiplying by 100. We observe a higher variability and skewness for WT biofilms both after 2 days and 6 days of formation, which are related to the high intrinsic variability of WT biofilm structure. This variability is based on the different observations that we used in our calculation of likelihood. The WT biofilm experimental data vary significantly across observations, whereas Mucoid and RSCV experimental data are relatively more comparable across observations. This intrinsic uncertainty (aleatoric uncertainty) is mainly due to the inherent randomness in WT biofilm dynamics, and it is different from the uncertainty (epistemic uncertainty) caused by the lack of enough experimental data.

**Table 2:** Coefficients of variation (CV) of estimated values for viscoelastic parameters of Burger model.

biofilm	elastic characteristics		viscosity characteristics	
	*E*_*m*_(%)	*E*_*k*_(%)	*η*_*m*_(%)	*η*_*k*_(%)
WT-2d	6.50	17.57	1.13	28.82
WT-4d	2.79	2.63	0.33	3.09
WT-6d	12.28	10.17	1.18	33.18
RSCV-2d	5.60	3.88	0.86	9.85
RSCV-4d	6.61	3.75	0.39	11.60
RSCV-6d	3.76	2.54	0.72	7.87
Mucoid-2d	8.83	2.51	0.41	6.30
Mucoid-4d	3.77	1.65	0.15	4.64
Mucoid-6d	2.87	3.73	0.76	3.65

**Table 3:** Skewness of estimated values for viscoelastic parameters of Burger model.

biofilm	elastic characteristics		viscosity characteristics	
	*E*_*m*_	*E*_*k*_	*η*_*m*_	*η*_*k*_
WT-2	1.89	−0.32	1.93	0.01
WT-4d	0.21	−0.11	0.21	0.01
WT-6d	1.10	0.29	0.50	1.02
RSCV-2d	0.49	0.16	0.31	0.20
RSCV-4d	0.54	0.12	0.13	0.34
RSCV-6d	0.33	0.11	0.13	0.19
Mucoid-2d	0.66	0.12	0.17	0.14
Mucoid-4d	0.28	0.08	0.05	0.12
Mucoid-6d	0.23	−0.16	0.33	−0.01

**Fig. 7:**
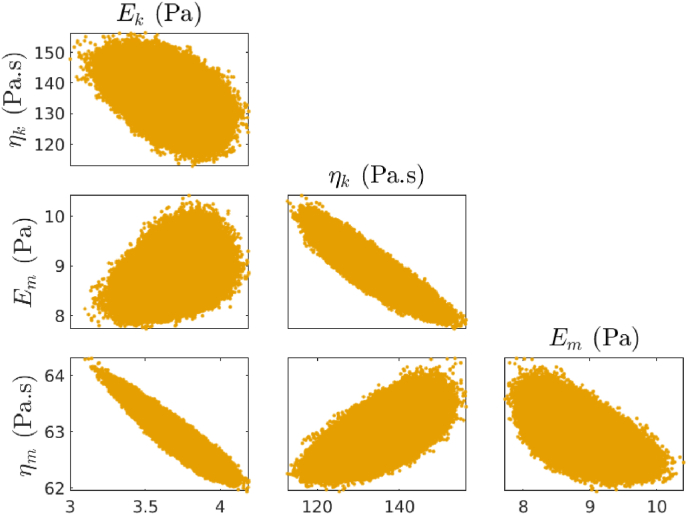
Correlation between all four viscoelastic parameters of WT after 4 days of formation.

Our Bayesian framework also provides us with the relationship between the parameters. Fig. 7 shows the correlation between parameters of WT biofilm after 4 days of formation as a triangle pair-wise plot. From this figure, we can conclude there is no direct relationship between *E*_*k*_, *η*_*k*_, *E*_*m*_, and *η*_*m*_. However, the relationship between *η*_*k*_ and *E*_*m*_ suggests that by increasing one parameter the other one decreases. The same correlation happens for *E*_*k*_ and *η*_*m*_.

**Fig. 8:**
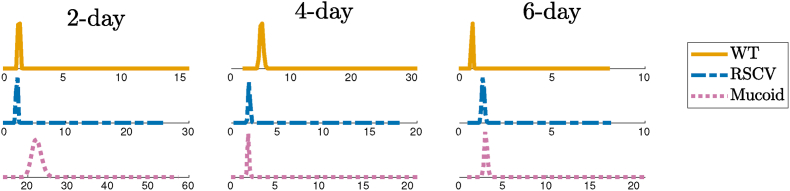
Posterior distribution of square root of error variance for WT, RSCV, and Mucoid after 2 days, 4 days, and 6 days of formation.

Then, we estimated the error variance in the prediction of the biofilm strain response by integrating the error variance as one of the parameters of interest in our Bayesian framework. Fig. 8 presents the probability density distributions for the square root of the error of variances *γ*. These results show that predicting the strain response for 2-day Mucoid is more difficult than other biofilm variants, mainly due to the missing data for the strain right before unloading the stress.

**Fig. 9:**
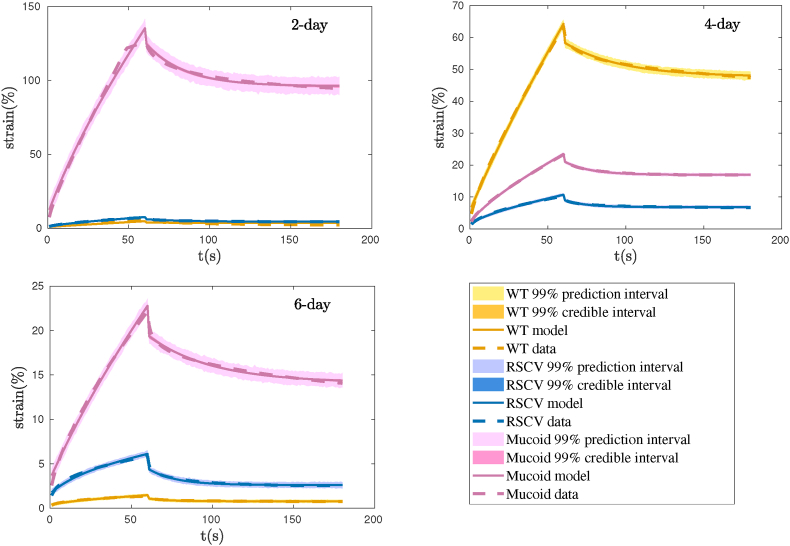
Prediction of strain vs the experimental data during the creep-recovery test for WT, RSCV, and Mucoid after 2 days, 4 days, and 6 days of formation.

After estimating the viscoelastic parameters as probability density functions, we used this information to predict the strain response. Assuming the mean behavior is representative, it can be used for a deterministic estimate of the viscoelastic parameters. The mean values of the posterior distributions were used to evaluate the model performance. The model prediction is compared with the experiments in Fig. 9. We observe that the Burger model is able to effectively quantify the strain response to the creep-recovery test for all the biofilms. As shown in Fig. 9, the strains due to the creep and recovery tests fit very well on the experimental data. However, at the end of the recovery part, there is a discrepancy between the model and data, which might be due to the complications with experiments. The propagation of uncertainties in the strain calculation is presented by displaying the 99% credible interval and 99% prediction intervals in the figure. These intervals are constructed based on the chains in Fig. 4. The 99% credible interval shows that after seeing the observed data with probability 99%, the strain is in the interval. However, in the calculation of the 99% prediction interval, error variance plays an important role and can predict future observations. The 99% prediction interval shows that after seeing the observed data with probability 99%, the strain of the future observation will be inside the plotted interval.

## 4 Discussion

Biofilms are subject to a wide range of shear forces over many magnitudes of time scales, many too short or too long for lab experimental test methods. Examples of these are high-speed interactions with water jets, such as interdental cleaning jets or pulse lavage in the wound and surgical site debridement as well as pressure washing of industrial surfaces such as ship hulls [48,49]. On the other hand, biofilms in the natural environment or on industrial surfaces are exposed to fluid forces over days to weeks to decades, impacting industrial performance. Predicting how biofilms may respond to these forces at time scales outside of normal testing methods will have application with respect to designing shear-based cleaning strategies and predicting long-term stability in systems such as uplift fermenters in wastewater and bioremediation systems.

Moreover, biofilms have repeatedly been shown to be highly variable making robust control methods very difficult [50]. One main outcome of this study is to demonstrate that, much of the variability in the mechanical properties of biofilms can be ascribed to variations in the microstructure that forms the EPS matrix. This understanding points to control strategies that target more specific components. This detailed information about the chemical structure of EPS components and an understanding of the impact of variations in the microstructure on the macroscopic behavior can lead to novel antibiofilm strategies.

**Fig. 10:**
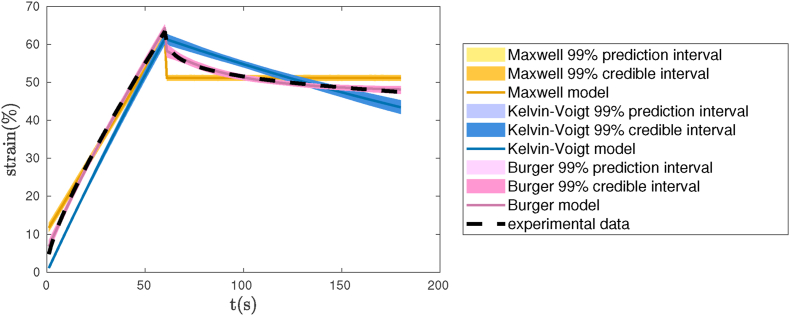
Comparison of Burger model against Maxwell and Kelvin-Voigt models, in strain prediction for WT after 4 days for formation.

The Burger viscoelastic model used in our study helped us obtain significantly better estimates for the viscosities and elasticities of our biofilm variants in comparison to the other well-known linear viscoelastic models, such as the Maxwell and Kelvin-Voigt, that are described in previous sections. This is mainly because the Burger model has the capability to describe instant elastic strain response, as well as time-dependent viscoelastic response and irrecoverable strain during a creep-recovery test. Fig. 10 shows the comparison of our predicted strain response using the Burger model for WT-4d biofilm, against the Maxwell and Kelvin-Voigt for the same biofilm variant. These strains were calculated based on the mean values of the viscoelastic parameters estimates using our Bayesian framework. The 99% credible and 99% prediction intervals are displayed in the figure to address the uncertainty in estimating the strain based on the given data as well as the uncertainty in the prediction of future observations based on the estimated parameters. The stochastic characteristics of our Bayesian framework helped us estimate the biofilm viscoelastic parameters with higher accuracy compared to existing models that used deterministic estimation techniques such as least-square fitting [40].

WT biofilms are very sensitive, and their mechanical properties vary significantly over time. First, the Maxwell and Kelvin-Voigt elasticities and viscosities decrease from day 2 to day 4, and then they increase. Biofilm elasticities and viscosities change over time and are less on day 4 than 2 before increasing by day 6. This is due to the higher affinity interactions between EPS components in 2-day and 6-day biofilms, compared to 4-day biofilms. Psl is known to be the dominant polysaccharide at the early stages of biofilm formation and makes the EPS matrix stiffer, whereas Pel is produced at later stages when the biofilm matures and makes the EPS matrix more viscous and malleable. These behaviors suggest the occurrence of different waves of EPS remodeling which results in elasticities and viscosities changes over time [24]. WT biofilms have very diverse components that lead to a large variation in mechanical properties. Presumably, this is because there are many ways that each biofilm can diversify the constituent production with relatively distinct properties. However, this allows for a larger signal-to-noise ratio than variants that overproduce one or more constituents. We observe RSCV biofilm elasticity to be almost constant over time. However, it is more viscous after 2 days and 6 days of formation. Mucoid biofilms, on the other hand, show a very low elasticity and viscosity at the first stages of formation, while as time goes by, they become more stiff and viscous. The biofilm mechanical properties are not subject to change after 4 days of formation, which shows their structural stability over time.

One interesting aspect of data assimilation techniques is their robustness with regard to cases where data is missing. In the context of creep-recovery experiments, extracting the biofilm strain response in the transition from stress loading (creep) and unloading (recovery) is challenging due to the rapid change in strain, experimental error, and the intrinsic nature of experiments that do not allow the operator to impulsively unload stress. This may result in low accuracy in quantifying the parameters of interest. Hence, the strain response experimental data for our 2-day WT biofilm was incomplete right before unloading, as it was difficult to capture the rapid drop in the strain. However, our data assimilation technique helped predict the unmeasured data and the strain for this time period of incomplete missing data.

## 5 Conclusion

In this paper, we have employed a mathematical framework to characterize the viscoelastic properties of *P. aeruginosa* biofilms during a creep-recovery test. We have described the strain response of WT *P. aeruginosa*, and isogenic RSCV and mucoid variant biofilms using a Burger viscoelastic model.

We have implemented an adaptive MCMC algorithm, that is based on a Bayesian estimation framework to estimate the model parameters based on the prior knowledge we have from the parameters and the experimental data. We have estimated the four model parameters involved in the viscoelastic constitutive equations for each biofilm after 2, 4, and 6 days of formation. The viscoelastic properties of these different biofilms are subject to a significant change over time, which shows the dynamic composition of the biofilm EPS structure. This type of study was pioneered in the early 2000s [40]. However, using a Bayesian framework and considering different strains have allowed us to incorporate recent advances in our understanding of biofilm mechanics. This analysis can help future research works elucidate the physics of the polymer network that forms the backbone of the biofilm [1]. This understanding is fundamental to the development of targeted therapies.

Additionally, addressing the fundamental variability of biofilm dynamics indicates weaknesses in the deterministic treatment of biofilm mechanics. Therefore, estimates of rheological properties using this method are more robust and descriptive than estimates using the geometry of relaxation curves. Our study also indicates that, since the properties of the constituents vary in time and density, methods to estimate the distribution between polymer types are needed.

This study contributes to our understanding of the connections between microscale structure and macroscale behavior. Additionally, we have demonstrated robust comparisons between our predictive model and experimental observations even in data sets with partial data. Modernizing our methodology and conceptualization of the impact of variable EPS microstructure encourages the development of highly targeted antibiofilm strategies. Understanding the underlying structure of biofilm and its impact on rheological properties provides novel directions to explore biofilm removal. For example, many biofilm removal techniques rely on applying forces to the biofilm to force sloughing [49]. By applying specific treatments that target different constituents, we can enhance this removal by manipulating the rheological properties. This requires a detailed understanding of the underlying distribution to optimize the targets.

The broad methodology investigated in this manuscript is directly applicable in many other settings. Developing tools to address the multi-component nature also plays a role when biofilms grow in soft matter such as within the mucus lining of the lungs in people with cystic fibrosis.

## Credit author Statement

M.N. derived model, implemented the numerical methods, wrote the manuscript. N.G.C. designed the study, contributed to analysis and writing the manuscript. P.S. and E.S.G. provided experimental data and biological insight, wrote the manuscript. M.Y.H. contributed funding for M.N. and participated in discussions regarding the research.

## Declaration of competing interest

The authors declare that they have no known competing financial interests or personal relationships that could have appeared to influence the work reported in this paper.

## Figures and Tables

**Figure:**
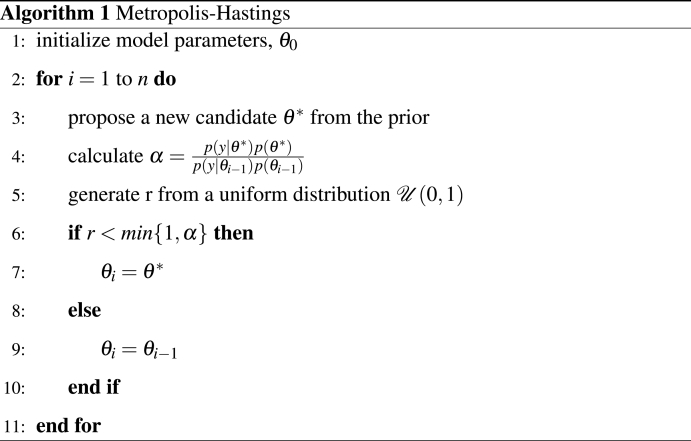


**Figure:**
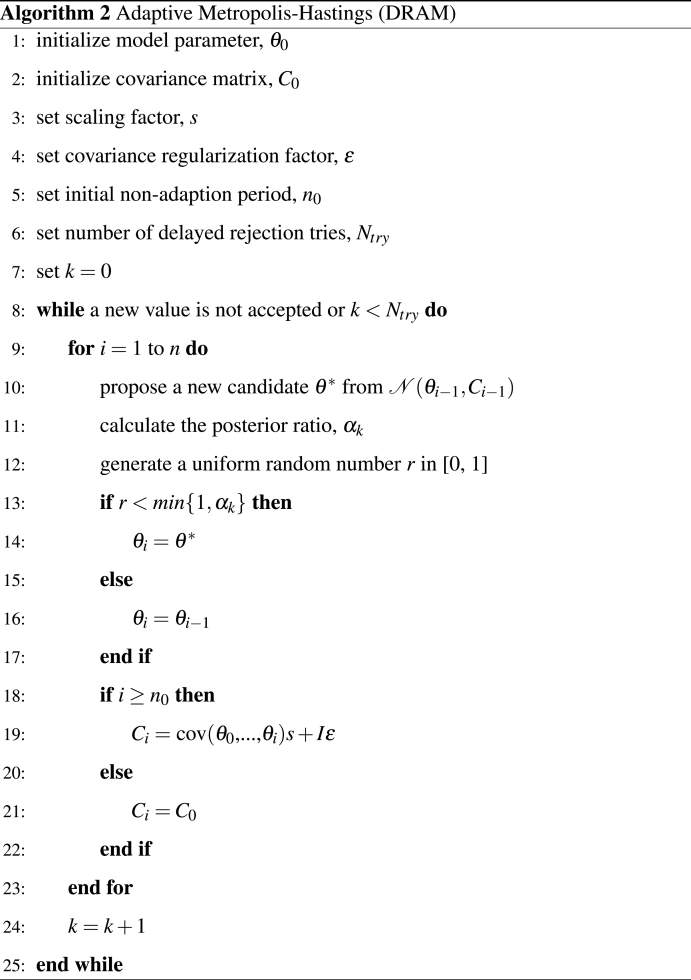


## Data Availability

Here is a link to our code used in this paper: https://github.com/nooranidoost/Bayesian_estimation_of_Pa_viscoelasticity

## Appendix A Numerical Algorithm for Parameter Estimation

In this Appendix, we provide the details of our Bayesian framework and the parameter estimation method. First, we explain Bayes’ theorem and the assumptions we considered to calculate the posterior distributions for a given set of model parameters. Then, we describe the Markov Chain Monte Carlo method and how to compute the target posterior distribution by evaluating the candidates that are sampled from a proposal distribution.

Bayes’ theorem is fundamental in the calculation of posterior distributions. In the case that the error variance *γ*^2^ is fixed and known, we can calculate the posterior of our physical model parameters *θ* based on the likelihood and prior of *θ*. Therefore, Bayes's theorem can be written as:

(A.1) $$p\left(\theta |y\right)=\frac{p\left(y|\theta \right)p\left(\theta \right)}{p\left(y\right)}$$

where *p*(*θ*|*y*) is our posterior distribution, the probability of model parameters given the observed data; *p*(*y*|*θ*) is the likelihood, the probability of observed data given the model parameters; and *p*(*θ*) is the prior distribution. The denominator *p*(*y*) is integral of the numerator over the parameter space, which is a normalization factor and is fixed. Note that, we do not need to compute the denominator as it cancels out in our calculations when we compare the sample candidates to decide whether reject or accept them.

In many scenarios, there is uncertainty in our data, and the error variance of parameters *γ*^2^ is unknown. This uncertainty can be quantified by integrating the error variance into our Bayesian framework [45]. In this case, the Bayes's theorem can be written as:

(A.2) $$p\left(\theta ,\gamma^{2}|y\right)=\frac{p\left(y|\theta ,\gamma^{2}\right)p\left(\theta ,\gamma^{2}\right)}{p\left(y\right)}$$

where *p*(*θ*, *γ*^2^|*y*) is our posterior distribution, the probability of model parameters and error variance given the observed data; *p*(*y*|*θ*, *γ*^2^) is the likelihood, the probability of observed data given the model parameters and error variance; and *p*(*θ*, *γ*^2^) is the joint prior distribution of *θ* and *γ*^2^. The i.i.d condition suggests that *p*(*θ*, *γ*^2^) = *p*(*θ*)*p*(*γ*^2^) because model parameters and error variance are independent parameters.

We sampled the model parameters *θ* from a uniform distribution with only positive numbers, as the physics of viscoelastic parameters do not allow them to take negative values. The error variance *γ*^2^ was sampled from an inverse *χ*^2^-squared distribution that is an uninformative conjugate prior to the normally distributed likelihood [45]. A Gaussian likelihood was employed to calculate the probability of the observed data given the model parameters. The Gaussian likelihood was chosen because we assumed our observations are mutually independent and identically distributed (i.i.d.), meaning that each observation has the same probability distribution as others, and the observations are mutually independent [43]. Thus, we assumed the errors are normally distributed with standard deviation *γ*, which is a common practice in many engineering and real-world applications [44]. Therefore, the likelihood, the probability of the observed data *y*, given the model parameters *θ* and the error variance *γ*^2^, can be written as:

(A.3) $$p\left(y|\theta ,\gamma^{2}\right)=\prod_{i=1}^{N}\frac{1}{\sqrt{\left(2\pi \right)}\gamma }e^{-\frac{{\left(x_{i}-y_{i}\right)}^{2}}{2\gamma^{2}}}$$

where *x*_*i*_, and *y*_*i*_ are the *i* th of the *N* model-derived estimates and observed data points, respectively.

Markov Chain Monte Carlo (MCMC) method generates sample candidates from a parameter space, that are either rejected or accepted based on the acceptance probability. Metropolis-Hastings (MH) is a classic sampling algorithm [46] (Algorithm 1) for Bayesian estimation. MH iteratively generates a sequence of sample candidates from a proposal distribution, in such a way that each sample is only dependent on the immediately preceding sample. Hence, it follows Markov Chain rules. Then, the sample candidates are either accepted or rejected based on how good the acceptance probability is compared to a uniform random number. Algorithm 1 Metropolis-Hastings In this paper, we employ the Delayed Rejection Adaptive Metropolis (DRAM) algorithm developed by Ref. [47] (Algorithm 2). This algorithm is a modified standard Metropolis-Hastings algorithm that helps improve the efficiency and speed of our computations by increasing convergence and acceptance rate. The idea behind Delayed Rejection (DR) is that, upon rejection in MH, instead of advancing time and retaining the same position, a second stage move is proposed, that can be extended to further proposal candidate sampling. Higher staged proposals are allowed to depend on the candidates already accepted or rejected, and their acceptance probabilities are dependent on the previous delayed rejection candidates [47]. Adaptive Metropolis (AM), unlike a regular MH algorithm, allows us to sample the posterior distribution based on the past samples’ path of the chain, which accelerates the convergence rate while keeping the ergodicity of the algorithm [47]. Algorithm 2 Adaptive Metropolis-Hastings (DRAM)
